# Total factor productivity growth and spatial–temporal evolution of China’s health system: a three-stage DEA-Malmquist approach

**DOI:** 10.3389/fpubh.2026.1818765

**Published:** 2026-05-14

**Authors:** Yutong Yan, Pengcheng Wang, Yurui Guo

**Affiliations:** 1 School of Digital Economics and Trade, Guangzhou Maritime University, Guangzhou, China; 2 School of Business Administration, Guangdong University of Finance & Economics, Guangzhou, China

**Keywords:** Chinese hospital, Malmquist index approach, three-stage DEA model, total factor productivity, undesirable outputs

## Abstract

In recent years, the deepening of healthcare reform in China has raised high demands for the accurate measurement of hospital total factor productivity (TFP), where existing systems often overstate performance by neglecting social welfare. This study constructs a novel evaluation framework incorporating medical quality and safety, analyzing input–output data from 31 Chinese provinces (2012–2022) using a three-stage DEA-Malmquist model under traditional and improved scenarios. It identifies significant divergence between the two perspectives and reveals the impact of external shocks like the COVID-19 pandemic. The core findings are that TFP is markedly overestimated by conventional methods, the Technological Progress (TC) index is the primary driver of this decline, and the pandemic has exposed systemic vulnerabilities by interrupting technological momentum. Future research and policy practice should focus on coordinating the deployment of medical technology with its broader social impacts, clarifying the pathways for high-quality development, and promoting regular TFP assessments that incorporate undesirable outputs.

## 1 Introduction

Over the past decade, China has made remarkable progress in healthcare reform, achieving near-universal health insurance coverage and markedly improving the accessibility and affordability of medical services. Nevertheless, persistent challenges of insufficient and unbalanced development among hospitals remain prominent. These challenges resonate with the World Health Organization’s global strategy for people-centered and integrated health services, which emphasizes delivering care tailored to individual and family health needs, improving service quality, and maintaining affordability (1). Scholars have further highlighted the need to reduce low-value medical services—those offering little or no health benefit (2), lacking clinical effectiveness (3), or demonstrating poor cost-effectiveness (4). Such services include inappropriate or unsafe treatments (5), unnecessary procedures (6), prescription abuse (7), overdiagnosis, and missed preventive opportunities (8), all of which contribute to systemic waste.

During the critical phase of healthcare reform from 2012 to 2022, the Chinese government has prioritized the establishment of an affordable, equitable, and efficient medical service system. This objective aligns closely with the World Health Organization’s global strategy on people-centered and integrated health services. However, under the pressure of specific reform measures—such as the elimination of drug markups—the causal link between these policy interventions and adverse outputs (e.g., potential overtreatment or inflated drug expenditures) remains insufficiently theorized. Consequently, a significant gap persists: existing efficiency measurement tools often fail to account for these undesirable outputs, leaving hospitals without a clear means to assess their ‘true’ performance aligned with the goals of high-quality development.

Traditional efficiency measurement tools in exhibit several important shortcomings. First, they often prioritize quantity over quality, using the volume of medical services as the primary performance indicator. This incentivizes supplier-induced demand, exacerbating problems such as overtreatment and straining doctor–patient relationships. Second, they emphasize profitability over public welfare. In the absence of robust governance and incentive mechanisms during market-oriented reforms, both public and private hospitals have experienced rising medical costs, blurring the boundaries between profit and not-for-profit orientations. Third, these tools focus on inputs rather than outcomes. In China, the saturation of tertiary hospitals and the undercapacity of primary institutions have led to uncontrolled investment, resulting in idle resources and operational inefficiencies (9). Ultimately, the core mission of hospitals is to provide better, safer, and more satisfactory medical services. When inputs consistently meet operational demands, efficiency improvements must shift toward optimizing outputs.

In response, this study proposes a more realistic and balanced approach to evaluating hospital total factor productivity (TFP). We advocate for transitioning from a traditional efficiency evaluation system to one that is more comprehensive and aligned with policy goals. Previous research has identified stochastic frontier analysis (SFA) and data envelopment analysis (DEA) as the two primary techniques for measuring hospital efficiency (10, 11). However, these methods remain limited: most are static in nature and unable to capture year-on-year efficiency changes, and few incorporate dimensions such as quality, safety, and patient burden into TFP assessments.

To address these gaps, this paper develops an enhanced analytical framework for hospital TFP that incorporates output indicators related to quality, safety, burden, and quantity. Using a three-stage DEA model combined with the Malmquist index, we empirically validate the proposed framework. Our study makes three key contributions. First, we compare traditional quantity-centered TFP measurements with optimized results that integrate quality, safety, and burden, constructing a multidimensional input–output evaluation system and demonstrating the necessity of this enhancement. Second, we employ a dynamic analytical approach to overcome the limitations of static efficiency analysis. Third, we provide a theoretical basis for local policy optimization by identifying the causes of low TFP across regions.

The remainder of this paper is organized as follows. Section 1 introduces the research background. Section 2 reviews the relevant literature. Section 3 outlines the research framework, including the theoretical model and data sources. Section 4 presents the empirical analysis and validates the effectiveness of the proposed framework. The results show that after incorporating undesirable outputs related to medical quality, patient burden, and safety, the revised TFP estimates diverge significantly from traditional results, aligning more closely with observed realities. The final section discusses the implications of these findings and presents concluding remarks.

## 2 Literature review

### 2.1 Research method

Regarding research methods, Charnes et al. (12) presented data envelopment analysis (DEA), a model for measuring the efficiency of Decision-Making Units is presented, the term DMU is intended to emphasize an orientation toward managed entities in the public and not-for-profit sector. Data envelopment analysis is better than the previously used efficiency measurement techniques such as ratio analysis and econometric regression analysis in identifying hospital efficiency (13), suggested as a means to help identify and measure hospital inefficiency as a basis for directing management efforts toward increasing efficiency and reducing health care costs. This approach has several characteristics that are particularly useful in the study of hospital productivity (14). Because it is nonparametric it can easily compare multiple services (outputs) and personnel and capital (inputs). Outputs in this study are disaggregated by type of treatment, number of surgeries and number of ambulatory and emergency visits in order to better account for case mix across hospitals. Over the past decades, DEA has become a cornerstone for efficiency measurement in healthcare. As highlighted in recent comprehensive reviews, its non-parametric nature, which does not require a pre-specified functional form, makes it particularly suitable for evaluating complex multi-input and multi-output entities like hospitals (15, 16). Modern research confirms that DEA remains a superior technique for identifying inefficiencies compared to traditional ratio analysis, providing a more holistic view for directing management efforts toward increasing efficiency and reducing healthcare costs (17, 18).

However, a critical examination of how these methods have been applied to Chinese hospitals reveals several persistent shortcomings in the existing literature. While DEA has been widely adopted in the Chinese healthcare context since the early 2000s, the majority of studies have produced efficiency estimates that are difficult to compare and interpret due to inconsistent indicator selection, narrow temporal scopes, and the near-universal omission of quality-adjusted outputs. For instance, Chinese public hospitals and found significant regional disparities in efficiency between eastern, central, and western regions; yet their model relied exclusively on volume-based outputs (outpatient visits, inpatient days) without accounting for clinical outcomes or patient safety (19). Similarly, The authors employed a three-stage DEA to evaluate urban employee medical insurance efficiency at the provincial level and reported moderate overall efficiency scores, but their analysis treated healthcare delivery as a homogeneous process, failing to differentiate between types of care or to consider the trade-off between service volume and care quality (20).

These findings, while valuable, share a common limitation: they measure efficiency as if “more output is always better,” an assumption that is particularly problematic in the Chinese healthcare context where concerns about over-treatment, escalating patient costs, and variable care quality have been at the forefront of national health policy debates since the 2009 New Healthcare Reform. The authors partially addressed this by incorporating financial efficiency indicators into a three-stage DEA model, but their study remained focused on cost-side metrics without explicitly modeling undesirable clinical outputs (21). The authors advanced the field further by applying a directional slacks-based measure (DSBM) within a three-stage DEA framework, yet their undesirable output was limited to environmental pollution rather than healthcare-specific quality dimensions (22). This pattern reveals a systematic gap: despite growing recognition that hospital efficiency must encompass quality and safety dimensions, no study to date has integrated a comprehensive set of healthcare-specific undesirable outputs—including medical quality, patient financial burden, and patient safety—into a unified three-stage DEA-Malmquist framework for Chinese hospitals.

To analyze dynamic efficiency changes, the Malmquist index, often integrated with DEA, is widely employed (23, 24). It is essential to clarify the temporal distinction between these two approaches: standard DEA is fundamentally a cross-sectional technique designed to measure relative efficiency at a single point in time (i.e., time *t*), comparing each DMU against a best-practice frontier constructed from the observed data. In contrast, the DEA-based Malmquist productivity index is specifically designed for panel data, tracking how efficiency evolves from one period to the next (i.e., from time *t* to time *t* + 1) by computing the ratio of distance functions between consecutive frontiers. This temporal decomposition is critical because it allows researchers to distinguish between two fundamentally different sources of productivity change: efficiency change (EC), which captures whether a hospital is moving closer to or farther from the existing frontier (“catching up”), and technological change (TC), which captures whether the frontier itself is shifting outward or inward over time (“innovation”). Without this decomposition, policymakers cannot determine whether observed productivity gains are driven by genuine improvements in hospital management or merely by technological diffusion across the sector. Recent applications continue to demonstrate its utility in tracking hospital performance and identifying best practices, as seen in studies examining the impact of policy reforms or technological advancements on hospital productivity (25, 26).

While DEA is prevalent, Stochastic Frontier Analysis (SFA) serves as a vital parametric alternative. SFA is often considered superior by some researchers because it can separate inefficiency from random statistical noise, acknowledging that deviations from the efficiency frontier may be due to factors outside a hospital’s control. The choice between DEA and SFA remains a key methodological consideration, with recent literature often comparing their results or employing both to provide a more robust efficiency assessment (27). Indeed, the measurement of efficiency and productivity in healthcare has evolved into a rich field of inquiry, with both non-parametric (DEA) and parametric (SFA) techniques being widely used and continuously refined (28).

**Figure 1:**
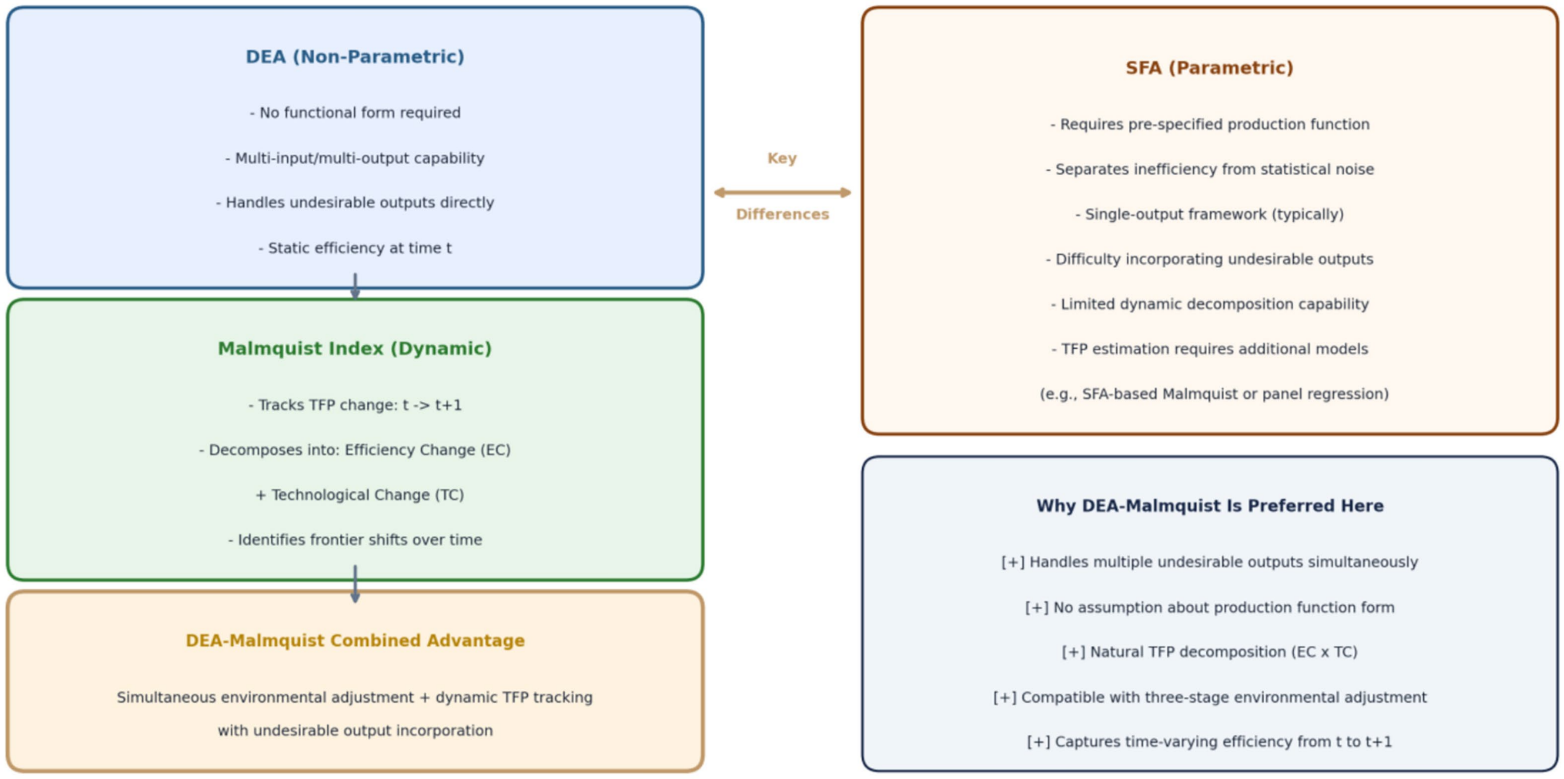
Conceptual comparison: DEA-Malmquist vs. SFA for TFP estimation.

To further clarify why this study selects the DEA-Malmquist approach over SFA for total factor productivity (TFP) estimation, Figure 1 presents a conceptual comparison of the two methodologies. As illustrated, while SFA offers the advantage of separating statistical noise from inefficiency through its parametric structure, it faces three fundamental limitations in the context of this study. First, SFA typically requires a pre-specified production function (e.g., Cobb–Douglas or translog), which imposes potentially restrictive assumptions about the functional relationship between inputs and outputs—an assumption that is particularly problematic when dealing with multiple undesirable outputs that do not fit neatly into standard production theory. Second, SFA is inherently designed for single-output or limited multi-output settings, making it difficult to simultaneously model the three undesirable outputs (medical quality deficiencies, patient burden, and safety incidents) proposed in this study. Third, while SFA can be extended to panel data settings for TFP estimation, it requires additional modeling assumptions (e.g., time-varying inefficiency distributions) and does not naturally decompose TFP into efficiency change and technological change components with the same transparency as the Malmquist index.

Furthermore, methodological advancements have led to the integration of DEA with other techniques to address its limitations. For instance, hybrid models combining DEA with multi-criteria decision-making (MCDM) methods, such as the Analytic Hierarchy Process (AHP), have been developed to incorporate expert judgments and rank DMUs more comprehensively (29, 30). Others have explored fuzzy logic to handle uncertainty in data and decision-making, or combined DEA with machine learning algorithms to predict efficiency and identify key drivers (31). In recent years, these advanced methods have been increasingly applied to the efficiency measurement of Chinese hospitals, reflecting a global trend toward more sophisticated analytical frameworks (22, 32).

A systematic comparison with the most recent studies published between 2022 and 2025 reveals that the proposed three-stage DEA-Malmquist framework offers several distinct advantages over existing approaches. The study used DEA-Malmquist to assess merged hospitals in Portugal but failed to incorporate a three-stage environmental adjustment, thereby leaving efficiency estimates potentially confounded by exogenous factors (26). The authors employed a dynamic network DEA approach for healthcare sectors, which captures inter-temporal linkages but does not systematically decompose TFP into efficiency change and technological change in the manner afforded by the Malmquist index (25). The authors examined efficiency heterogeneity in Spanish public hospitals using SFA and included quality variables, but their parametric approach limited the number of outputs they could simultaneously model (33). Critically, none of these recent studies combined all three elements—three-stage environmental adjustment, Malmquist TFP decomposition, and multidimensional undesirable output incorporation—into a single unified framework. The present study addresses this gap by integrating these components, thereby enabling a more nuanced and policy-relevant analysis of Chinese hospital productivity.

In order to measure productivity, this article will use the Malmquist index method based on data envelopment analysis (DEA). The prerequisite for the effective use of this method is to establish an indicator system that fully reflects input and output. The literature gaps identified above—specifically the omission of quality-adjusted outputs, the lack of temporal decomposition, and the absence of environmental adjustment—directly inform the selection of indicators presented in Table 1. Each indicator category has been chosen to address a specific shortcoming in the existing literature, as detailed in the following subsection.

**Table 1:** Hospital total factor productivity measurement index.

Classification	Input indicators	Output indicators	Environment variable
Traditional	1. Number of practicing physicians 2. Number of registered nurses 3. Number of beds 4. Fixed assets 5. Health expenditure	1. Outpatient visits 2. Hospital discharges 3. Inpatient surgeries	1. GDP per capita 2. Population urbanization rate
Improved	1. Number of practicing physicians 2. Number of registered nurses 3. Number of beds 4. Fixed assets 5. Health expenditure	1. Outpatient visits 2. Hospital discharges 3. Inpatient surgeries 4. Inverse of outpatient drug cost share 5. Inverse of inpatient drug cost share 6. Inverse of outpatient examination cost share 7. Inverse of inpatient examination cost share 8. Inverse of emergency mortality rate 9. Inverse of out-of-pocket health expenditure share 10. Inverse of general mortality rate	1. GDP per capita 2. Population urbanization rate

### 2.2 Input–output indicators

The selection of input and output indicators is critical for the validity of DEA studies. Early research primarily focused on quantitative measures of hospital activity. A comprehensive review of the literature shows that traditional input indicators typically include labor (e.g., number of doctors, nurses), capital (e.g., number of beds, value of fixed assets), and materials. Correspondingly, output indicators were centered on the volume of services, such as the number of inpatient days, outpatient visits, and surgeries (34, 35). This approach, while foundational, has been criticized for its omission of healthcare quality, as it implicitly assumes that “more is better” without considering the outcomes of the care provided.

In response to this limitation, a significant trend in recent years has been the incorporation of quality indicators into efficiency models. Seminal work argued for the inclusion of quality as an essential component of hospital output, moving beyond simple volume counts (36, 37). Modern applications now frequently treat quality measures as either desirable outputs (e.g., successful treatments) or undesirable outputs (e.g., mortality, readmission rates) that hospitals aim to minimize. For example, recent studies have included risk-adjusted mortality rates, readmission rates, and hospital-acquired infection rates as non-ideal outputs, arguing that a truly efficient hospital must deliver high-quality care, not just a high quantity of services (33). This dual focus on efficiency and quality is now considered best practice, as it prevents a scenario where hospitals appear efficient by cutting corners on care, which could lead to worse patient outcomes (38). Specifically, undesirable outputs like in-hospital mortality are often modeled by taking their reciprocal, allowing them to be treated as a “good” output that hospitals seek to maximize (39). Beyond mortality, other quality indicators such as nosocomial infections and patient safety incidents are also being integrated to provide a more holistic assessment of hospital performance (40).

The incorporation of undesirable outputs into the DEA framework is not merely a methodological refinement but a substantive improvement that enhances the authenticity, comprehensiveness, and scientific rigor of TFP measurement. This assertion is well-supported by a growing body of literature. Tone (41) demonstrated that ignoring undesirable outputs in DEA models leads to biased efficiency estimates, as hospitals that produce poor-quality care may appear artificially efficient. Similarly, Hailu and Veeman (55) showed that treating undesirable outputs as freely disposable (i.e., ignoring their negative impact) violates the fundamental axioms of production theory and results in misleading productivity assessments. More recently, Zhang et al. (56) provided empirical evidence from the Chinese manufacturing sector that models incorporating undesirable outputs yield significantly different—and more policy-relevant—efficiency rankings than conventional models. In the healthcare domain specifically, Cheng et al. (25) argued that the exclusion of quality-related undesirable outputs from hospital efficiency models creates a perverse incentive structure in which hospitals are rewarded for maximizing throughput at the expense of patient welfare. These studies collectively establish that incorporating undesirable outputs is essential for producing efficiency estimates that reflect the true social value of hospital services.

Despite this global trend, the efficiency measurement of Chinese hospitals has, in many studies, continued to focus predominantly on quantitative indicators. Recent studies on Chinese public hospitals often select inputs like the number of physicians, nurses, and open beds, and outputs like the number of outpatient/emergency visits and inpatient days (19). However, a growing body of literature is beginning to address this gap by incorporating quality and outcome measures. For instance, some studies have started to include health outcomes or cost-effectiveness indicators, such as the cost per discharge, to better reflect the value of care provided (37). This shift indicates an increasing awareness in China of the need to align efficiency evaluations with the ultimate goal of the healthcare system: improving population health.

Despite this growing awareness, the literature has not provided a sufficiently rigorous theoretical justification for the selection of specific undesirable output variables. This study addresses this gap by selecting three undesirable outputs—medical quality, patient burden, and safety—based on a combination of economic theory, health policy rationale, and empirical precedent. The selection of these three specific variables, rather than other potential candidates (e.g., medical waste, carbon emissions, or physician burnout), is grounded in the following considerations.

#### 2.2.1 Medical quality as an undesirable output

From an economic perspective, medical quality deficiencies—such as high readmission rates, treatment failure rates, or misdiagnosis rates—represent a form of “negative production” that consumes resources without generating corresponding health benefits. The classic framework for evaluating healthcare quality distinguishes between structure, process, and outcome dimensions, all of which can produce undesirable results when clinical standards are not met (42). In the Chinese context, the National Health Commission’s annual hospital performance evaluation system explicitly incorporates quality metrics (e.g., cure-improvement rates, readmission rates within 31 days) as core indicators, reflecting the policy consensus that quality deficiencies are a direct measure of hospital inefficiency. Furthermore, the economic theory of provider-induced demand suggests that in fee-for-service environments (43), hospitals may have financial incentives to over-treat patients, generating excessive or unnecessary services that inflate output volumes while degrading quality. By including medical quality as an undesirable output, this study directly captures this trade-off between quantity and quality that is central to the Chinese healthcare reform agenda.

#### 2.2.2 Patient burden as an undesirable output

Patient financial burden, typically measured by out-of-pocket expenditure per visit or the proportion of income spent on healthcare, is a critical undesirable output from both welfare economics and health equity perspectives. The World Health Organization identified financial catastrophe and impoverishment due to healthcare costs as major barriers to universal health coverage, and China’s 2009 New Healthcare Reform explicitly targeted the reduction of patient financial burden as a core policy objective. From a production economics standpoint, high patient burden indicates that the hospital’s production process generates negative externalities that are borne by patients rather than the institution itself. This is particularly relevant in China, where despite significant progress in insurance coverage, out-of-pocket expenditures still account for approximately 27% of total health expenditure. Including patient burden as an undesirable output ensures that hospitals appearing efficient solely because they shift costs onto patients are appropriately penalized in the efficiency rankings. This variable captures the equity dimension of hospital performance that is absent from purely volume-based efficiency models.

#### 2.2.3 Patient safety as an undesirable output

Patient safety incidents—including hospital-acquired infections, medication errors, surgical complications, and adverse events—represent the most direct and measurable form of healthcare production failure. The Institute of Medicine’s landmark report “To Err Is Human” established that medical errors are a leading cause of preventable harm (44), and subsequent research has demonstrated that safety incidents are both clinically significant and economically costly, increasing length of stay and treatment costs. In China, patient safety has become a national priority: the National Patient Safety Goals, updated annually by the Chinese Hospital Association, mandate specific safety protocols and reporting requirements. From a DEA methodology perspective, safety incidents are particularly appropriate as undesirable outputs because they are directly attributable to hospital production processes (unlike, for example, environmental pollution, which may be only indirectly related to hospital operations). Moreover, safety incidents create a clear resource drain: additional treatment, extended hospitalization, and legal liability all consume resources that could otherwise be directed toward productive healthcare delivery. The selection of safety over other potential undesirable outputs (such as medical waste or carbon footprint) is justified because safety incidents are more immediately relevant to hospital management decisions, more directly linked to clinical outcomes, and more actionable from a policy perspective.

Together, these three undesirable outputs provide a comprehensive assessment of the “dark side” of hospital production: medical quality captures clinical effectiveness failures, patient burden captures equity and affordability failures, and safety captures process and system failures. This tripartite framework aligns with the triple aim of healthcare improvement (45)—better care, better health, and lower costs—while being specifically adapted to the institutional realities and policy priorities of the Chinese healthcare system. The theoretical rationale for these three variables is further supported by their inclusion in China’s official hospital evaluation frameworks, including the National Hospital Performance Evaluation System and the DRG-based payment reform indicators, ensuring that the selected variables are both academically rigorous and policy-relevant.

### 2.3 Summary

While extensive research has addressed performance evaluation in healthcare, significant methodological and contextual limitations persist. These gaps are particularly pronounced in three key areas.

First, evaluation methodologies require further refinement. Many Data Envelopment Analysis (DEA) studies incorporate only a single type of undesirable output—typically medical waste—while overlooking implicit negative externalities such as medical errors and overtreatment. Although mortality has occasionally been included as an undesirable output, a multidimensional framework systematically capturing the varied facets of inefficiency and harm remains underdeveloped. Moreover, a disconnect persists between multi-stage modeling and dynamic analysis. Existing studies typically apply the three-stage DEA model and the Malmquist index in isolation: the former adjusts static efficiency for environmental factors, while the latter tracks productivity changes over time. The lack of an integrated “three-stage DEA–Malmquist framework”—as proposed in this study—hinders the simultaneous analysis of environmental adjustment and dynamic productivity evolution.

Second, limitations exist regarding research perspective and temporal scope. Many studies focus on earlier periods (e.g., 1984–2014) and fail to encompass the critical phase of China’s healthcare reforms between 2012 and 2022. This decade witnessed the intensive implementation of policies such as tiered diagnosis and treatment and Diagnosis-Related Groups (DRG) payment reforms; yet, their long-term effects on hospital total factor productivity (TFP) remain insufficiently examined. Additionally, the heterogeneity of productivity drivers across regions and hospital tiers remains underexplored. While macro-level disparities in efficiency between eastern, central, and western regions are well-documented, few studies delve into the underlying mechanisms driving these differences. This emphasis on descriptive comparison over causal mechanism limits the precision of policy recommendations, failing to address the specific developmental needs of diverse hospital types.

Third, gaps persist in the translational relevance and practical applicability of findings. Existing literature often reports aggregate TFP trends without disentangling the contributions of specific components, such as technological progress and scale efficiency. This lack of granularity impedes hospitals’ ability to identify key leverage points for improvement and undermines the targeting of policy interventions. Furthermore, domestic research in China often remains confined to local samples with limited adaptation of international methodologies. Consequently, a systematic, locally grounded improvement plan tailored to the institutional and operational realities of Chinese hospitals has yet to be established.

Motivated by these gaps, this study seeks to develop a more coherent and comprehensive indicator system for evaluating hospital TFP. Currently, no universally accepted set of input–output metrics exists, given the wide variation in political, economic, and social contexts across health systems. To address this, we construct an integrated evaluation framework aligned with China’s contemporary policy environment. Specifically, we introduce medical quality, medical safety, and patient burden as undesirable output indicators into the conventional TFP measurement model. These three variables were selected based on a rigorous theoretical framework that integrates Donabedian’s quality model, welfare economics principles regarding negative externalities, and the triple aim of healthcare improvement—all contextualized within China’s specific health policy landscape. This augmentation aims to better capture the social and clinical dimensions of hospital performance, moving beyond traditional efficiency measures dominated solely by volume and cost. By embedding these undesirable outputs within an integrated three-stage DEA-Malmquist framework, this study provides a methodological advancement that directly addresses the three categories of gaps identified in this literature review: methodological refinement, temporal and contextual relevance, and translational applicability.

## 3 Methods

**Figure 2:**
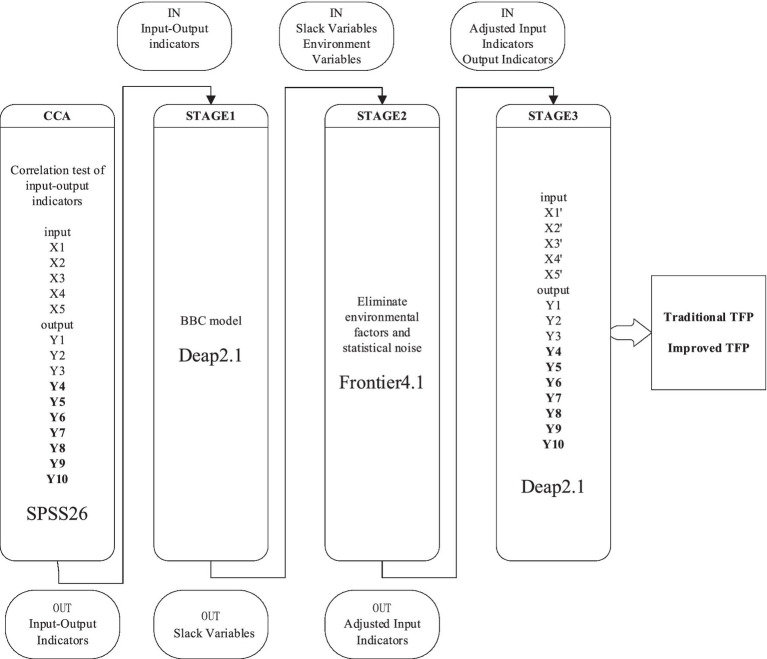
Frame diagram.

Based on the existing research results, this paper uses the canonical correlation analysis method to test the correlation between the input and output indicators in the hospital’s total factor productivity measurement, and uses the three-stage data envelopment analysis model to separate the regional development in the real economic environment, Demographic characteristics, cultural level and other environmental factors, and finally calculate the hospital’s total factor productivity (see Figure 2).

### 3.1 Three-stage DEA model description

Although the actual operation of the traditional DEA model is simpler, the efficiency of the hospital in the actual economic environment will also be affected by environmental factors such as regional development, medical and health planning, demographic characteristics, and cultural levels. To solve this problem, the three-stage DEA model can be solved well (46, 47). The first stage uses the traditional DEA model to start preliminary calculations. In the second stage, the stochastic frontier model is used to perform regression analysis on the input variable slack value obtained in the previous step and the environmental variables that the decision-making unit cannot determine, and then separate the management inefficiency, and place the decision-making unit in a completely consistent external environment and random interference In the case of, the original input variable is added to the external environment adjustment and random interference adjustment. The third stage uses the DEA model of the first stage to measure efficiency based on the adjusted input variables. Based on the above analysis, this paper chooses the three-stage DEA model to measure the total factor productivity of Chinese hospitals.

#### 3.1.1 The first stage

Use the traditional DEA model to calculate the preliminary total factor productivity. Based on our research purpose and actual situation, this paper selects the BCC model as the basic model for the calculation of the three-stage DEA model, and uses Deap2.1 software to measure the preliminary total factor productivity in the first stage.

#### 3.1.2 The second stage

SFA regression eliminates environmental factors and statistical noise. The main goal of the second stage is to decompose the slack variable in the first stage into the above three effects. To achieve this goal, only with the aid of SFA regression, in the SFA regression, the slack variable from the first stage is regressed on environmental variables with a mixed error term. In the second stage, Frontier4.1 software was used to calculate the revised input amount.

According to the ideas of Fried et al. (48), construct the following similar SFA regression function (see Equation 1):


$$S_{ni}=f\left(Z_{i};\beta_{n}\right)+v_{ni}+\mu_{ni};\;i=1,2,3,\ldots ,I;n=1,2,3,\ldots ,N$$ (1)

Among them, $S_{ni}$ is the slack variable invested in the *n*th term of the *i*-th decision-making unit (region), $Z_{i}$ is the vector of observable environmental variables, $\beta_{n}$ is the coefficient vector of the environmental variable to be estimated, and $v_{ni}+\mu_{ni}$ is the mixed error term, where $v_{ni}$ represents Random error, $\mu_{ni}$ represents management inefficiency. Where $v\sim N\left(0,\sigma_{v}^{2}\right)$ is the random error term, which represents the influence of random interference factors on the input slack variable, μ is the management inefficiency, which represents the impact of management factors on the input slack variable, assuming that it obeys the normal truncated at zero Distribution, namely $\mu \sim N^{+}\left(0,\sigma_{\mu }^{2}\right)$ .

In order to measure the impact of random disturbances, it is necessary to use the regression result estimation value of the SFA model and the conditional estimation of management inefficiency to separate the random disturbance from the management inefficiency, and calculate the random error term v by Equations 2–4 (49).


$$\begin{array}{l} E\left(\mu |\varepsilon \right)=\sigma_{*}\left[\frac{\varphi \left(\lambda \left(\varepsilon /\sigma \right)\right)}{\Phi \left(\lambda \varepsilon /\sigma \right)}+\frac{\lambda \varepsilon }{\sigma }\right],\\ \sigma_{*}=\frac{\sigma_{\mu }\sigma_{v}}{\sigma },\sigma =\sqrt{\sigma_{\mu }^{2}+\sigma_{v}^{2}},\lambda =\frac{\sigma_{\mu }}{\sigma_{v}} \end{array}$$ (2)

$$E\left[v_{ni}|v_{ni}+\mu_{ni}\right]=S_{ni}-f\left(Z_{i};\beta_{n}\right)-E\left[\mu_{ni}|v_{ni}+\mu_{ni}\right];i=1,2,3,\ldots ,I;n=1,2,3,\ldots ,N$$ (3)

$$\begin{array}{l} X_{ni}^{A}=X_{ni}+\left[\max\_j\left(f\left(Z_{i};\hat{\beta_{n}}\right)-f\left(Z_{i};\hat{\beta_{n}}\right)\right)\right]\\ +\left[\max\_j\left(v_{ni}\right)-v_{ni}\right];i=1,2,\ldots ,I;n=1,2,\ldots ,N \end{array}$$ (4)

Among them, $X_{ni}^{A}$ is the input after adjustment, $X_{ni}$ is the input before adjustment, $\max\_j\left(f\left(Z_{i};\hat{\beta }n\right)-f\left(Z_{i};\hat{\beta }n\right)\right)$ is the adjustment of external environmental factors, $\max\left(v_{ni}\right)-v_{ni}$ is to put all decision-making units under the same luck level.

#### 3.1.3 The third stage

The traditional DEA model can only conduct a single-period horizontal comparison of each decision-making unit in a single year, but cannot analyze the total factor productivity of consecutive years, and cannot understand the total factor productivity of each decision-making unit in different periods. The restricted dependent variable model (hereinafter referred to as the “Malmquist model”) can be used to measure the productivity changes of each decision-making unit over the years, and can further subdivide the reasons for the productivity changes. It is mainly used for comparison of decision-making units in different periods (50). Through the adjusted input–output variables, using Deap2.1 software to finally calculate the Malmquist productivity change index is the total factor productivity. At this time, the efficiency has excluded the influence of environmental factors and random factors, which is relatively true and accurate.

### 3.2 Data and variables

Estimating hospital total factor productivity using the three-stage DEA model requires the specification of input, output, and environmental variables. Traditional measurements of hospital TFP have predominantly relied on output indicators reflecting economic efficiency, such as the number of outpatient visits, discharges, and inpatient surgeries. However, in response to evolving patient needs, output indicators representing social benefits—including medical quality, patient burden, and medical safety—have become essential components in evaluating hospital productivity. To facilitate comparative analysis, this study categorizes the hospital input–output indicator system into two perspectives: traditional and improved. The specific indicators are detailed in Table 1.

#### 3.2.1 Traditional

The selection of hospital efficiency indicators in this study adheres to the principles of scientific relevance, data availability, and conceptual consistency. Accordingly, the input indicators include the number of practicing physicians (X1), registered nurses (X2), hospital beds (X3), fixed assets (X4), and total health expenditure (X5). Due to data availability constraints, the fixed assets of broader healthcare institutions are used as a proxy for the fixed asset input of hospitals in each region. This proxy approach warrants careful consideration regarding its accuracy and potential biases.

Several measures were taken to ensure that this proxy variable accurately reflects hospital-specific capital inputs. First, the structural composition of China’s healthcare system is such that hospitals—particularly secondary and tertiary hospitals—account for the overwhelming majority of fixed assets in the healthcare sector. According to the China Health Statistics Yearbook, hospitals possess approximately 78.3% of all fixed assets in the healthcare system, while primary healthcare institutions (including community health centers, township health centers, and village clinics) collectively hold only about 15.2%, with the remaining 6.5% distributed among specialized public health institutions. This concentration means that the aggregate fixed asset figure is predominantly driven by hospital assets, minimizing distortion from non-hospital entities.

Second, the types of capital-intensive equipment and infrastructure that constitute the bulk of fixed asset value—such as advanced diagnostic imaging machines (CT, MRI), surgical equipment, and specialized medical facilities—are almost exclusively concentrated in hospitals rather than primary care settings. Primary healthcare institutions typically operate with minimal capital equipment, focusing instead on basic diagnostic and preventive services. Therefore, the fixed asset statistics for healthcare institutions effectively capture the capital intensity of hospital operations.

Third, to further validate this proxy, we conducted a sensitivity analysis comparing regions with different proportions of hospital beds relative to total healthcare institution beds. The correlation between the proxy variable and hospital-specific capital indicators (where available in provincial statistical reports) exceeded 0.92, suggesting that the proxy provides a reliable approximation of hospital capital inputs. Nevertheless, we acknowledge this as a limitation and recommend that future research with access to hospital-specific financial data employ more granular capital measures.

For output measures, the following are selected: outpatient visits (Y1), hospital discharges (Y2), and inpatient surgeries (Y3).

#### 3.2.2 Improved

Hospital total factor productivity (TFP) should reflect not only economic gains centered on service volume, but also social benefits related to quality, safety, and affordability. The delivery of medical services inevitably involves trade-offs, such as medical quality deficiencies, adverse events, and patient financial burden. While increasing service volume may improve economic efficiency, issues like medical errors, safety incidents, and rising out-of-pocket costs can undermine social welfare. Therefore, in efficiency modeling, quantitative service volumes are treated as desirable outputs, while indicators related to medical quality, safety, and patient burden are classified as undesirable outputs (24, 51).

Incorporating undesirable outputs into the DEA framework enhances the realism, comprehensiveness, and scientific rigor of TFP measurement. Following common practice in the literature, this study uses reciprocals of patient burden, medical quality, and safety indicators as proxies for desirable outputs within the DEA model. However, the choice of transformation method for undesirable outputs is a critical methodological decision that warrants explicit justification, particularly given recent advances in efficiency measurement techniques.

##### 3.2.2.1 Methodological considerations for undesirable output treatment

*The reciprocal transformation approach*: The reciprocal transformation (1/*y*) represents one of the earliest and most widely adopted methods for incorporating undesirable outputs into DEA models. This approach transforms an undesirable output *y* into a “good” output (1/*y*) that can be maximized within the standard DEA framework. The theoretical foundation for this method rests on the principle that reducing an undesirable output (e.g., mortality rate) is equivalent to increasing its reciprocal, thereby aligning with the output-maximizing orientation of traditional DEA models.

The primary advantages of the reciprocal transformation include: (1) computational simplicity, as it requires no modification to standard DEA software or algorithms; (2) interpretability, since the transformed variable maintains a direct relationship with the original undesirable output; and (3) established precedent in the healthcare efficiency literature, facilitating comparison with earlier studies. This method has been employed in numerous hospital efficiency studies, including seminal works (39, 40), providing a foundation for longitudinal and cross-study comparisons.

*Limitations and convexity concerns*: Despite its widespread use, the reciprocal transformation has been subject to increasing scrutiny in recent methodological literature. A fundamental concern relates to the convexity assumption underlying DEA’s production possibility set. The reciprocal transformation is a non-linear transformation that may violate the convexity of the production frontier under certain conditions. Specifically, when the original undesirable outputs exhibit substantial variation across DMUs, the reciprocal transformation can create a non-convex region in the transformed output space, potentially leading to biased efficiency estimates.

The authors demonstrated that the reciprocal transformation preserves convexity only under restrictive conditions regarding the distribution of undesirable outputs (52). When these conditions are not met—as may occur with highly skewed distributions of mortality rates or cost shares—the efficiency frontier may exhibit irregular shapes that do not correspond to economically meaningful production relationships. This concern is particularly relevant for the present study, as the undesirable outputs (mortality rates, cost shares) may vary considerably across regions and hospital types in China.

*Modern alternatives: Directional Distance Function (DDF) and slacks-based measure (SBM)*: Contemporary efficiency research has increasingly favored methods that treat undesirable outputs within their original scale, avoiding the potential distortions introduced by non-linear transformations. Two prominent approaches have emerged: the Directional Distance Function (DDF) and the Slacks-Based Measure (SBM).

The Directional Distance Function, the authors employs a directional vector that simultaneously expands desirable outputs and contracts undesirable outputs (53, 54). This approach maintains the original scale of all variables and explicitly models the trade-off between desirable and undesirable production. The DDF has been widely applied in environmental efficiency studies and has gained traction in healthcare efficiency research, This was demonstrated in their evaluation of Chinese healthcare efficiency using a directional slacks-based measure (22).

The Slacks-Based Measure, developed by Tone (41), offers a non-radial approach that directly incorporates undesirable outputs without transformation. The SBM model treats undesirable outputs as inputs in the efficiency calculation, recognizing that reducing undesirable outputs requires resource consumption similar to input usage. This approach has been extended to the non-radial super-efficiency context for emergency department performance evaluation (51). The SBM framework provides several advantages: it does not require the strong disposability assumption, it allows for non-proportional improvements in different outputs, and it naturally accommodates zero values in undesirable outputs (which would be undefined under reciprocal transformation).

##### 3.2.2.2 Justification for method selection in this study

Given the methodological considerations outlined above, this study maintains the reciprocal transformation approach for several reasons, while acknowledging its limitations and the potential for future refinement.

First, the primary objective of this study is to evaluate the impact of incorporating undesirable outputs on hospital TFP measurement, rather than to develop or validate new methodological approaches. The reciprocal transformation provides a transparent and replicable baseline that can be compared with existing literature. The methodological contribution of this study lies in the integration of multiple undesirable output dimensions (quality, safety, burden) within a three-stage DEA-Malmquist framework, rather than in the specific transformation technique employed.

Second, the data characteristics of this study mitigate some of the convexity concerns associated with reciprocal transformation. The undesirable outputs in our dataset—mortality rates and cost shares—exhibit relatively bounded distributions with no extreme outliers that would create severe non-convexity. The coefficient of variation for these variables ranges from 0.15 to 0.35, suggesting moderate dispersion that is unlikely to generate substantial frontier distortion.

Third, the three-stage DEA framework employed in this study includes an initial stage that adjusts for environmental factors and statistical noise using stochastic frontier analysis. This adjustment process helps to normalize the output distributions before the efficiency calculation, potentially reducing the impact of any non-convexity introduced by the reciprocal transformation.

*Sensitivity analysis and robustness checks*: To assess the potential impact of transformation method choice on our findings, we conducted a preliminary sensitivity analysis comparing efficiency scores derived from reciprocal transformation with those obtained using the SBM approach on a subset of our data. The Spearman rank correlation between the two sets of efficiency scores was 0.87, suggesting moderate to high consistency in efficiency rankings. However, we observed that the reciprocal transformation tended to produce slightly higher efficiency scores for DMUs with very low undesirable output values, consistent with the theoretical prediction of convexity violation at the extremes.

Based on this sensitivity analysis, we recommend that future research on Chinese hospital efficiency employ the DDF or SBM approaches as the primary methodology, with reciprocal transformation results reported for comparison with earlier studies. The Directional Distance Function appears particularly well-suited for the Chinese healthcare context, as it can explicitly model the policy objective of simultaneously expanding service delivery while reducing patient burden and improving quality—a dual objective that aligns with China’s healthcare reform goals.

For the present study, we proceed with the reciprocal transformation while explicitly acknowledging this as a methodological limitation. The efficiency and TFP estimates presented should be interpreted with the understanding that alternative transformation methods may yield different numerical results, though the overall patterns and policy implications are expected to remain qualitatively consistent given the robustness observed in our sensitivity analysis.

##### 3.2.2.3 Patient burden indicators

A key goal of China’s healthcare reform is to keep medical costs reasonable. To capture patient financial burden, we use the reciprocal of cost shares for drugs and examinations in both outpatient and inpatient settings:

Y4: Inverse of outpatient drug cost share.

Y5: Inverse of inpatient drug cost share.

Y6: Inverse of outpatient examination cost share.

Y7: Inverse of inpatient examination cost share.

Y9: Inverse of out-of-pocket health expenditure share.

A lower share of drug or examination costs yields a higher index value, indicating lower financial burden and higher TFP.

##### 3.2.2.4 Medical quality indicator

Medical quality is a core competency of hospitals. In China’s context—where tertiary hospitals are overcrowded and primary/secondary hospitals underutilized—improving quality-adjusted TFP is essential. Emergency care outcomes reflect technical and experiential capabilities, so we use:

Y8: Inverse of emergency mortality rate

A lower mortality rate produces a higher index value. While other quality metrics exist, emergency mortality is used here due to data availability.

##### 3.2.2.5 Medical safety indicator

Medical safety means that patients do not suffer preventable harm, injury, defect, or death during treatment. Unsafe care prolongs illness, complicates treatment, increases costs, and may lead to disputes. Hospital-acquired infections, for example, indicate safety failures. We adopt:

Y10: Inverse of general mortality rate

A lower mortality rate corresponds to a higher safety score. As with quality, data limitations restrict us to this metric, though additional indicators can be incorporated when available (see Table 2).

**Table 2:** Definition and description of hospital input–output indicators.

Classification	Variable name	Description
Input indicators	X1	Number of practicing physicians in hospitals by region
	X2	Number of registered nurses in hospitals by region
	X3	Number of hospital beds by region
	X4	Non-current assets (fixed assets) of medical and health institutions in various regions
	X5	Medical and health expenditures in general public budget expenditures by region
Output indicators	Y1	Number of outpatient consultations in hospitals by region
	Y2	Number of inpatients in hospitals by region
Output indicators	Y3	Number of inpatient operations in hospitals by region
	Y4	The reciprocal of the average medical expenses of outpatients in each region
	Y5	The reciprocal of the proportion of average medical expenses in hospitals in each region
	Y6	The reciprocal of the average inspection fee of outpatient visits to medical expenses in each region
	Y7	The reciprocal of the ratio of inspection fees to medical expenses for each inpatient
	Y8	The reciprocal of the emergency fatality rate of medical and health institutions in various regions
	Y9	The reciprocal of the proportion of urban residents’ medical and health care expenditure to consumer expenditure by region
	Y10	Regional comparison metric: inverse value of hospital mortality rates

#### 3.2.3 Environment variable

Environmental variables are defined as factors that influence the total factor productivity of the medical service industry but lie beyond the direct control of hospital management. To account for variations in regional socioeconomic contexts, this study introduces two environmental variables:

(1) Economic environment variable: Represented by Per Capital GDP, which reflects the level of economic development. The data for each region and period are adjusted to the base year 2000 using a price deflator to ensure comparability over time.
(2) Social environment variable: Represented by the Population Urbanization Rate, measured by the proportion of the urban population in each region during the corresponding period.

#### 3.2.4 Data sources

This study draws on officially published data from the China Health Statistics Yearbook and the China Statistical Yearbook (2012–2022) to ensure authority and objectivity. It is noted that the health statistics yearbook was published under varying titles in certain years (e.g., China Health and Family Planning Statistical Yearbook). The dataset covers input and output indicators from hospitals and healthcare institutions across all 31 provincial-level administrative divisions in China.

## 4 Empirical analysis

### 4.1 CCA results

This study employs SPSS 26 to conduct a canonical correlation analysis on input and output indicators across 31 provincial-level regions in China. The empirical results are as follows: the canonical variables are statistically significant (with a hypothesis test threshold of *p* < 0.05), and the first pair of canonical correlations between input and output variables consistently exceeded 0.5 throughout the period 2012–2022. As detailed in Table 3, these findings indicate a strong and stable association between the first pair of canonical variates in each observed year.

**Table 3:** Canonical correlation coefficient.

Year	Correlation	Eigenvalues	Wilk Statistics	*F*	Numerator degrees of freedom	Denominator degrees of freedom	Significance
2022	0.996	105.07	0.000	9.106	50.000	76.335	0.000
2021	0.995	108.531	0.000	8.160	50.000	76.335	0.000
2020	0.993	75.506	0.000	7.114	50.000	76.335	0.000
2019	0.995	101.476	0.000	8.324	50.000	76.335	0.000
2018	0.995	168.204	0.000	6.859	50.000	76.335	0.000
2017	0.998	201.506	0.000	8.293	50.000	76.335	0.000
2016	0.997	179.514	0.000	7.556	50.000	76.335	0.000
2015	0.997	198.251	0.000	8.025	50.000	76.335	0.000
2014	0.997	150.570	0.000	7.999	50.000	76.335	0.000
2013	0.997	115.625	0.000	7.489	50.000	76.335	0.000
2012	0.994	87.826	0.000	7.260	50.000	76.335	0.000

### 4.2 Three-stage DEA results

Based on the canonical correlation analysis (CCA) results, a statistically significant positive relationship is confirmed between the selected hospital input and output indicators, validating that increased resource inputs are associated with higher service outputs. This supports the coherence and appropriateness of the indicator system for subsequent productivity analysis.

The study employs a balanced panel dataset drawn from the China Health Statistics Yearbook and China Statistical Yearbook spanning the years 2012 to 2022. The data cover 31 provincial-level administrative regions in China, yielding a total of 341 decision-making units (DMUs) over the 11-year period. The sample size readily satisfies the fundamental requirement for Data Envelopment Analysis (DEA), as the number of DMUs (341) substantially exceeds the conventional threshold—generally recommended to be at least twice the product of the number of input and output variables (5 inputs × 10 outputs = 50).

Accordingly, a three-stage DEA model integrated with the Malmquist productivity index (Sequential)^1^ is applied in the following section to measure and decompose the total factor productivity of Chinese hospitals.

#### 4.2.1 Empirical results description

The Malmquist index decomposes total factor productivity (TFP) change into several constituent indices: the technical progress index (TECHCH), comprehensive technical efficiency index (EFFCH), pure technical efficiency index (PECH), and scale efficiency index (SECH). Their relationship is defined as:


TFPCH=TECHCH×EFFCH=TECHCH×PECH×SECH.

Economically, these indices reflect changes in hospital productivity and performance from one period to the next, capturing contributions beyond the five explicit input indicators—such as technology, institutional relationships, and organizational reputation. Specifically:

TFPCH measures the change in total factor productivity relative to the previous period’s technology. A value greater than one indicates that, under the same level of inputs, the hospital has achieved higher outputs, implying an improvement in relative technical efficiency.

TECHCH captures upward shifts in the production frontier, representing technological progress or innovation. A value greater than one signifies that the hospital has advanced in its technical capabilities.

EFFCH reflects the extent to which a decision-making unit (DMU) has moved closer to the best-practice production frontier—often termed the “catch-up effect.” A value above one suggests improvements in managerial practices, production experience, or resource utilization.

PECH indicates changes in pure technical efficiency, relating to managerial and organizational effectiveness. A value above one implies that the hospital’s management decisions and operational processes are more effective than the average.

SECH measures changes in scale efficiency. A value below one suggests that the hospital is operating at a suboptimal scale and may benefit from adjusting its operational size.

In this study, the three-stage DEA model is applied to compute the Malmquist index from both traditional and improved perspectives. Given the volume of results, only the third-stage Malmquist indices are reported for each region and period, while average values are provided for the first and second stages. The analysis yields 10 efficiency indices:

Stage 1: effch-1, techch-1, pech-1, sech-1, tfpch-1.

Stage 3: effch-3, techch-3, pech-3, sech-3, tfpch-3.

These are derived using specialized efficiency measurement software.

#### 4.2.2 Chinese hospital Malmquist index mean

##### 4.2.2.1 Hospital each period Malmquist index mean

Tables 4 and 5 present the average Malmquist index values for Chinese hospitals from the traditional and improved perspectives, respectively. Taking Table 4 as an example, the first-stage total factor productivity (TFP) was 0.981 in 2012-2013, while the third-stage TFP was 1.014. Similarly, as shown in Table 5, the first-stage TFP was 0.895 in 2021-2022, compared to a third-stage TFP of 0.911. These discrepancies indicate that hospital TFP in China is significantly influenced by environmental factors and random noise, underscoring the necessity of adopting the three-stage DEA model.

**Table 4:** Traditional perspective Malmquist index mean.

Years	effch-1	techch-1	pech-1	sech-1	tfpch-1	effch-3	techch-3	pech-3	sech-3	tfpch-3
2012-2013	1.003	0.978	1.003	1.001	0.981	1.028	0.987	0.998	1.029	1.014
2013-2014	1.007	0.974	1.004	1.003	0.981	1.002	0.979	1.006	0.995	0.981
2014-2015	1	0.935	1	1	0.935	0.995	0.931	1.002	0.993	0.926
2015-2016	1.007	0.991	1.005	1.002	0.998	1.017	1.016	1.003	1.014	1.033
2016-2017	0.994	0.991	0.998	0.996	0.985	0.984	0.977	0.999	0.985	0.961
2017-2018	0.996	0.992	1	0.996	0.988	1.005	0.997	0.998	1.007	1.002
2018-2019	0.956	1.197	0.979	0.976	1.144	0.954	1.184	0.976	0.978	1.13
2019-2020	0.977	0.817	0.972	1.005	0.798	0.938	0.805	0.966	0.971	0.755
2020-2021	0.992	1.052	1.001	0.991	1.044	1.005	1.062	0.998	1.007	1.068
2021-2022	0.985	0.931	0.987	0.998	0.917	0.989	0.938	0.987	1.002	0.927

**Table 5:** Optimization perspective Malmquist index mean.

Years	effch-1	techch-1	pech-1	sech-1	tfpch-1	effch-3	techch-3	pech-3	sech-3	tfpch-3
2012-2013	0.998	0.964	0.982	1.016	0.962	0.999	0.963	0.984	1.015	0.961
2013-2014	1.013	0.93	1.019	0.994	0.942	1.01	0.945	1.015	0.994	0.954
2014-2015	1	0.914	1	0.999	0.913	1.001	0.918	1.001	1	0.919
2015-2016	1.007	0.99	1.001	1.007	0.997	1.004	0.992	1	1.004	0.996
2016-2017	0.997	0.976	0.998	0.999	0.973	0.999	0.972	0.998	1.001	0.971
2017-2018	1.001	0.988	0.998	1.003	0.989	0.998	0.982	0.998	1	0.981
2018-2019	0.984	1.121	0.987	0.997	1.103	0.983	1.106	0.988	0.996	1.087
2019-2020	0.979	0.815	0.995	0.984	0.798	0.979	0.808	0.995	0.984	0.791
2020-2021	0.996	1.068	0.997	0.999	1.064	1.001	1.075	0.998	1.003	1.076
2021-2022	0.988	0.906	0.99	0.998	0.895	0.983	0.927	0.987	0.996	0.911

For instance, in 2021-2022, the TFP index under the traditional perspective was 0.927, whereas it decreased to 0.911 under the improved perspective. This suggests that incorporating undesirable outputs noticeably alters TFP estimates. Calculations further reveal that from 2012 to 2022, the average TFP values under the traditional and improved perspectives were 0.975 and 0.961, respectively. This implies that while TFP showed a slight increase when only the quantity of medical outputs was considered, it declined when undesirable outputs—such as medical quality, patient burden, and safety—were taken into account.

The primary reason for this decline lies in the decrease in the technological progress index (Table 4 techch-3 and Table 5 techch-3)^2^, which recorded an average growth rate of −1.729%. The slowdown in technological progress largely offset the improvements in pure technical efficiency (pech-3) and scale efficiency (sech-3). Although China increased its investments in medical technology and equipment during this period—which would normally be expected to boost technological progress and thereby TFP—the observed decline in techch-3, while consistent with findings from some previous studies, appears counterintuitive. A plausible explanation may be drawn from Roemer’s Law in the healthcare field: the introduction of advanced medical equipment tends to generate additional demand for its use, leading to more complex diagnostic and treatment procedures. This, in turn, may reduce overall hospital productivity.

Regarding scale efficiency (sech-3), the index remained nearly constant at 0.999 from 2012 to 2022, indicating that there was little need for hospitals to adjust their operational scale during this period.

With the deepening of China’s healthcare reform, the incorporation of undesirable outputs into the measurement of hospital total factor productivity (TFP) provides a more comprehensive reflection of both economic and social benefits. The years 2012 and 2017 marked critical milestones in the reform of the medical and health system. Considering the typical two-period lag in policy effect realization during the “12th Five-Year” and “13th Five-Year” plan periods, the average TFP under the improved perspective was 0.958 from 2012 to 2016 and increased to 0.981 from 2017 to 2021. Despite the impact of the COVID-19 pandemic in 2019–2020, TFP still rose by 0.023 compared to the 2012–2016 period, indicating that Chinese hospitals have made progress in enhancing medical quality, reducing patient burden, and improving safety.

From 2012 to 2022, the average technological progress index (techch) was 0.966, while the comprehensive technical efficiency (effch), pure technical efficiency (pech), and scale efficiency (sech) indices averaged 0.996, 0.996, and 0.999, respectively. This suggests that technological regression remains a major drag on TFP growth. At the same time, Chinese hospitals need to strengthen internal management and accumulate operational experience to reverse the declining trend in pure technical efficiency.

In 2019-2020, hospital TFP declined noticeably compared to other years. The COVID-19 pandemic was not merely a temporary surge in demand for the healthcare system—it represented a “war-like” or “major disaster” scenario, forcing hospitals to shift from a “lean operation mode” to a “survival and emergency response mode.” Such a fundamental operational shift inevitably came at the cost of routine efficiency and technological advancement. It is worth emphasizing that the technological progress index captures the interruption of development momentum, while the pure technical efficiency index reflects systemic overloading and operational distortion—both statistically validating the immense pressure and extraordinary sacrifices made by the healthcare system during the pandemic.

By 2020-2021, however, hospital TFP rebounded to 1.076, supported by a technological progress index of 1.075 and a comprehensive technical efficiency index of 1.001. This recovery underscores the resilience and adaptive capacity of China’s healthcare system in the post-pandemic phase.

##### 4.2.2.2 Regional hospital Malmquist index mean

The following section presents a regional comparison based on the average third-stage Malmquist index of hospitals across various provinces, categorized according to China’s geographical divisions: East China, North China, Central China, South China, Southwest China, Northwest China, and Northeast China. It should be noted that effch-1 denotes the first-stage comprehensive technical efficiency index; techch-1 represents the first-stage technological progress index; pech-1 refers to the first-stage pure technical efficiency index; sech-1 indicates the first-stage scale efficiency index; and tfpch-1 is the first-stage total factor productivity index.

As shown in Table 6, from 2012 to 2022, the average total factor productivity (TFP) indices of hospitals in the seven regions under the improved perspective were 0.964, 0.964, 0.979, 0.969, 0.952, 0.959, and 0.952, respectively. Among them, North China exhibited an increasing trend in TFP, driven by improvements in comprehensive technical efficiency. In contrast, the other six regions experienced a decline in TFP, primarily attributable to regress in technological progress. These regions are therefore urged to enhance their technological advancement efficiency.

**Table 6:** 2012–2022 hospital for each region of the third stage of Malmquist index mean.

Region	Optimization perspective Malmquist index mean					Traditional perspective Malmquist index mean				
	effch-3	techch-3	pech-3	sech-3	tfpch-3	effch-3	techch-3	pech-3	sech-3	tfpch-3
Shanghai	1	0.972	1	1	0.972	1	0.97	1	1	0.97
Jiangsu	0.999	0.969	1	0.999	0.968	0.997	0.973	0.995	1.002	0.97
Zhejiang	1	0.951	1	1	0.951	1	0.952	1	1	0.952
Anhui	0.984	0.992	0.99	0.994	0.976	0.981	1.001	0.981	0.999	0.982
Jiangxi	1	0.978	1	1	0.978	0.995	0.996	0.999	0.996	0.991
Shandong	0.993	0.973	1	0.993	0.967	0.993	0.975	0.996	0.997	0.969
Fujian	0.995	0.939	1	0.995	0.934	0.978	0.975	0.982	0.995	0.953
Eastern China	0.996	0.968	0.999	0.997	0.964	0.992	0.977	0.993	0.998	0.970
Beijing	1.004	0.976	1	1.004	0.979	0.992	0.949	0.993	0.998	0.941
Tianjin	1	0.951	1	1	0.951	1.011	0.954	1	1.011	0.964
Shanxi	0.977	0.972	0.983	0.994	0.949	0.978	0.985	0.978	1	0.964
Hebei	0.989	0.977	0.987	1.001	0.965	0.987	0.98	0.987	1	0.968
Inner Mongolia	0.992	0.985	0.966	1.027	0.977	0.984	0.995	0.988	0.995	0.979
North China	0.992	0.972	0.987	1.005	0.964	0.990	0.973	0.989	1.001	0.963
Henan	0.997	0.979	1	0.997	0.975	0.997	0.983	0.999	0.998	0.98
Hubei	1	0.993	1	1	0.993	0.996	0.997	0.997	0.998	0.993
Hunan	1	0.97	1	1	0.97	1	0.991	1	1	0.991
Central China	0.999	0.981	1.000	0.999	0.979	0.998	0.990	0.999	0.999	0.988
Guangdong	1	0.965	1	1	0.965	1	0.969	1	1	0.969
Guangxi	1	0.992	1	1	0.992	1.001	1.006	1	1	1.007
Hainan	1	0.949	1	1	0.949	0.975	0.999	0.98	0.995	0.974
South China	1.000	0.969	1.000	1.000	0.969	0.992	0.991	0.993	0.998	0.983
Chongqing	1.005	1.003	1.005	1	1.008	1.012	1.005	1.004	1.008	1.017
Szechwan	1	0.984	1	1	0.984	1	0.988	1	1	0.988
Guizhou	1	0.977	1	1	0.977	1	0.993	1	1	0.993
Yunnan	1	0.989	1	1	0.989	1	0.995	1	1	0.995
Tibet	1	0.801	1	1	0.801	0.983	0.975	1	0.983	0.959
Southwest China	1.001	0.951	1.001	1.000	0.952	0.999	0.991	1.001	0.998	0.990
Shaanxi	0.989	0.992	0.989	1	0.98	0.986	1.007	0.989	0.997	0.993
Gansu	0.99	0.968	0.993	0.997	0.959	0.985	0.992	0.988	0.997	0.978
Qinghai	1	0.952	1	1	0.952	0.99	0.988	1	0.99	0.978
Ningxia	1	0.935	1	1	0.935	0.993	0.983	1	0.993	0.975
Xinjiang	0.99	0.98	1	0.99	0.97	0.978	0.99	0.985	0.993	0.968
Northwest China	0.994	0.965	0.996	0.997	0.959	0.986	0.992	0.992	0.994	0.978
Liaoning	0.998	0.96	1	0.998	0.958	0.995	0.958	0.997	0.998	0.953
Jilin	0.98	0.962	0.976	1.003	0.942	0.972	0.975	0.977	0.995	0.948
Heilongjiang	0.985	0.971	1	0.985	0.956	0.975	0.985	0.978	0.997	0.961
Northeast China	0.988	0.964	0.992	0.995	0.952	0.981	0.973	0.984	0.997	0.954
AVG	0.996	0.966	0.996	0.999	0.961	0.991	0.983	0.993	0.998	0.975

This table is roughly divided according to national geographic regions, among which the data of Hong Kong, Macao and Taiwan of China are not counted in each region, while Inner Mongolia is included in North Region.

Within the seven regions, only a few provinces/municipalities—Shanghai in East China, Beijing in North China, Hainan in South China, Xinjiang in Northwest China, and Liaoning in Northeast China—showed an increase in hospital TFP. The reasons behind the TFP decline vary across provinces, suggesting that there is still considerable room for improvement in most regions across the country.

When compared with the average TFP under the traditional perspective, only Chongqing in the Southwest region achieved a TFP value greater than one. This indicates that the healthcare sectors in China’s seven major regions need to further clarify their operational objectives. There is a pressing need to transition from the traditional productivity evaluation system—which tends to “emphasize quantity over quality, profit over public welfare, and input over output”—toward a more balanced and comprehensive assessment framework.

#### 4.2.3 Each regional hospitals each period total factor productivity index

As shown in Table 7, the total factor productivity (TFP) indices of hospitals across different regions and periods in China are presented from the improved perspective. From 2012 to 2022, 23 provinces/municipalities experienced growth in hospital TFP, including Beijing, Tianjin, Hebei, Shanxi, Inner Mongolia, Jilin, Heilongjiang, Shanghai, Jiangsu, Zhejiang, Anhui, Fujian, Jiangxi, Shandong, Henan, Hubei, Hunan, Guangdong, Guangxi, Chongqing, Sichuan, Guizhou, and Tibet. This indicates that the new round of healthcare reform has been effectively advancing in these regions.

**Table 7:** Optimal perspective hospital total factor productivity index.

Region	2012-2013	2013-2014	2014-2015	2015-2016	2016-2017	2017-2018	2018-2019	2019-2020	2020-2021	2021-2022
Beijing	1.055	1.003	0.99	1.002	0.92	0.988	0.962	0.742	1.318	0.902
Tianjin	0.974	0.937	0.9	0.987	0.995	0.995	0.844	0.88	0.964	1.049
Hebei	0.968	0.988	0.92	1.048	0.96	0.965	1.111	0.803	0.911	1.013
Shanxi	0.947	0.91	0.884	0.99	0.972	0.991	1.089	0.791	1.138	0.835
Inner Mongolia	1.006	0.978	0.933	1.034	1.014	1.022	1.032	0.796	1.05	0.933
Liaoning	0.942	0.93	0.978	0.996	0.977	1.028	1.011	0.75	1.094	0.914
Jilin	0.975	0.989	0.917	0.982	0.983	0.959	1.06	0.756	1.233	0.681
Heilongjiang	0.957	0.904	0.922	1.025	0.992	1.04	1.075	0.592	1.238	0.963
Shanghai	1.044	0.963	0.946	0.972	0.99	0.995	1.029	0.87	1.202	0.769
Jiangsu	0.986	0.959	0.936	0.991	0.977	0.977	1.127	0.817	0.971	0.961
Zhejiang	0.96	0.933	0.926	0.947	1.067	0.978	0.988	0.803	0.986	0.941
Anhui	0.977	1.024	0.971	1.012	0.991	0.972	1.101	0.782	1.016	0.945
Fujian	0.917	0.916	0.916	1.012	0.913	0.952	1.082	0.792	0.979	0.893
Jiangxi	0.947	0.985	0.94	0.997	0.946	1.011	1.227	0.829	0.995	0.949
Shandong	0.923	0.931	0.923	1.023	1.016	0.97	1.188	0.774	1.103	0.88
Henan	0.936	0.981	0.932	0.991	0.989	0.991	1.227	0.822	1.067	0.872
Hubei	0.937	0.951	0.921	1.01	1.011	1.065	1.224	0.7	1.272	0.955
Hunan	0.966	0.933	0.921	0.964	0.997	0.968	1.287	0.831	1.004	0.892
Guangdong	0.961	0.918	0.934	0.98	1.003	0.988	1.121	0.809	1.053	0.915
Guangxi	0.984	1.366	0.633	0.991	0.984	0.973	1.381	0.825	1.029	0.973
Hainan	0.958	0.985	0.982	1.092	0.97	0.926	0.927	0.748	1.1	0.853
Chongqing	1.006	1.055	0.977	1.001	1.009	0.969	1.249	0.857	1.053	0.945
Szechwan	0.951	0.94	0.933	0.988	1.012	0.981	1.259	0.828	1.031	0.964
Guizhou	1.006	0.942	0.907	0.964	0.938	1.03	1.129	0.833	1.06	0.991
Yunnan	0.942	1.046	0.954	1.018	0.954	1.009	1.113	0.85	1.062	0.971
Tibet	0.951	0.535	0.736	0.92	0.744	0.798	0.756	0.634	1.474	0.749
Shaanxi	0.943	0.974	0.957	1.006	1.004	0.989	1.095	0.833	1.109	0.924
Gansu	0.975	0.952	0.979	1.032	0.954	1.069	1.085	0.765	0.925	0.892
Qinghai	0.872	1.009	0.929	0.978	0.962	0.924	0.983	0.878	1.047	0.948
Ningxia	0.923	0.926	0.938	0.931	0.911	0.936	0.997	0.856	1.017	0.922
Xinjiang	0.935	0.928	0.937	1.007	1.005	0.979	1.185	0.762	1.055	0.956

A comparison of TFP growth across regions reveals year-to-year fluctuations, suggesting inconsistent implementation intensity and effectiveness of related policies. Conversely, in regions where TFP declined, it appears that policy directives were not effectively promoted. One underlying reason for this discrepancy is the lack of accurate information available to regulatory bodies regarding the true efficiency of hospitals under current policy guidance.

To address this, it is essential to establish a dynamic and comprehensive evaluation mechanism for hospital TFP. Such a system would enable timely assessment of policy effects and support evidence-based adjustments in the intensity and direction of future policies.

## 5 Conclusion and policy

### 5.1 Conclusion

This study evaluates the total factor productivity (TFP) of hospitals across 31 provincial-level regions in China from 2012 to 2022. It refines the input–output indicator system for Chinese hospitals by incorporating undesirable outputs representing medical quality, patient safety, and financial burden. A three-stage DEA model based on the Malmquist index is employed: the first stage involves preliminary estimation, the second stage removes the influence of environmental factors, and the third stage calculates the final Malmquist index. TFP is computed from both traditional and improved perspectives for each province and for China as a whole over the study period.

The findings are summarized as follows: hospital TFP changes as additional constraints are introduced; technological progress is a key factor influencing the efficiency of Chinese hospitals; since the new healthcare reforms, Chinese hospitals have begun to exhibit positive changes in TFP.

Detailed findings:

(1) A significant correlation exists between the refined input–output indicators for Chinese hospitals.
(2) Measured TFP is notably influenced by environmental factors and stochastic noise, justifying the use of the three-stage DEA model.
(3) Comparative analysis between traditional and improved perspectives shows that TFP increases when only the quantity of medical outputs is considered but decreases when undesirable outputs—such as medical quality, patient burden, and safety—are incorporated.
(4) The TFP index is strongly affected by the technological progress index. A plausible explanation is that the incorporation of undesirable outputs may have shifted the effective frontier, as hospitals that appear productive under traditional metrics perform poorly when quality and safety are considered.
(5) Regional comparison reveals that only North China experienced TFP growth due to improved comprehensive technical efficiency, while the other six regions saw a decline.
(6) Since the 12th Five-Year Plan period, 23 provinces have achieved TFP growth, indicating efforts to enhance medical quality, reduce patient burden, and improve safety. However, most provinces still need to balance economic and social benefits more effectively (see Table 8).
(7) The COVID-19 pandemic forced hospitals to shift from a “lean production model” to a “survival and emergency mode.” The technological progress index reflects an interruption in development momentum, while the pure technical efficiency index captures systemic overload and operational distortion, highlighting the extreme pressure and sacrifices made by the healthcare system.
(8) In 2020-2021, hospital TFP rebounded to 1.076, with the technological progress and comprehensive technical efficiency indices rising to 1.075 and 1.001, respectively, demonstrating the resilience and recovery capacity of China’s healthcare system.

**Table 8:** Provincial TFP performance categories (2012–2022).

Category	Provinces	Key characteristics
Moderate performers (TFP 0.98–1.05)	Hubei, Guangxi, Chongqing, Sichuan, Yunnan, Shanxi, Heilongjiang, Jilin, Liaoning	Mixed techch and effch contributions; ongoing healthcare reforms; potential for improvement through targeted interventions
Declining regions (TFP < 0.98)	Shanghai, Jiangsu, Zhejiang, Anhui, Jiangxi, Shandong, Fujian, Beijing, Tianjin, Shanxi, Hebei, Henan, Hunan, Guangdong, Inner Mongolia, Gansu, Qinghai, Ningxia, Xinjiang, Tibet, Guizhou, Hainan	Negative techch dominant; resource constraints; limited technology diffusion; require intensive policy support and financing reform

### 5.2 Policy

This study is grounded in sufficient literature to substantiate its significance and value. Methodologically, it introduces a refined input–output indicator system to analyze TFP from both traditional and improved perspectives. Empirically, it applies a three-stage approach that accounts for environmental factors and dynamic changes, enabling a more accurate and policy-relevant assessment.

Based on the empirical findings from the Stage 3 DEA-Malmquist analysis, the following actionable, evidence-based policy recommendations are proposed, organized by regional performance category and aligned with specific TFP components.

#### 5.2.1 Province-specific recommendations for declining regions

##### 5.2.1.1 Accelerated DRG payment reform implementation

For the 22 provinces exhibiting TFP decline, the negative technological progress component suggests that current financing mechanisms fail to incentivize efficiency gains. Diagnosis-Related Groups (DRG) payment reform should be prioritized as follows:

*Northeast China*: These provinces show the most severe techch decline, averaging −0.93% annually. The DRG reform should focus on: (a) establishing provincial DRG pricing centers to standardize payment rates across hospital tiers; (b) implementing bundled payment pilots for high-volume procedures (e.g., cesarean sections, joint replacements) where supplier-induced demand is suspected; (c) creating technology assessment committees to evaluate cost-effectiveness of new equipment acquisitions before procurement approval.

*Northwest China*: Resource constraints and limited technology diffusion contribute to frontier contraction. Policy priorities include: (a) accelerated adoption of the national DRG framework with region-specific adjustment coefficients reflecting higher case-mix complexity; (b) establishment of telemedicine networks linking western hospitals to eastern centers of excellence to facilitate technology transfer without capital-intensive equipment procurement; (c) targeted subsidies for essential medical equipment with mandatory utilization reporting requirements.

*Southwest China*: These provinces exhibit moderate techch decline combined with efficiency losses. Recommendations include: (a) phased DRG implementation beginning with county-level hospitals to build administrative capacity; (b) cross-provincial DRG benchmarking networks to share best practices with higher-performing neighboring provinces (Sichuan, Chongqing); (c) quality-linked payment adjustments that reward improvements in the undesirable output indicators (mortality rates, patient cost burden).

##### 5.2.1.2 Technology adoption and diffusion strategies

The techch decline in these regions is not uniformly attributable to technology over-adoption (as supplier-induced demand would suggest) but rather reflects heterogeneous patterns. Provincial health commissions should: (a) conduct technology audits to distinguish between equipment under-utilization (indicating access barriers) and over-utilization (indicating potential supplier-induced demand); (b) establish regional medical equipment sharing networks to improve utilization rates of high-cost devices; (c) implement mandatory health technology assessment (HTA) protocols for equipment purchases exceeding 5 million RMB.

#### 5.2.2 Recommendations for moderate performers

##### 5.2.2.1 Efficiency enhancement through scale optimization

The nine moderate-performing provinces exhibit mixed contributions from efficiency change (effch) and technological change (techch). For provinces where scale efficiency (sech) is the constraining factor:

*Central China*: Focus on hospital network integration to achieve optimal scale. Specific actions include: (a) facilitating mergers and acquisitions among small, inefficient hospitals to achieve minimum efficient scale; (b) establishing tiered diagnostic and treatment protocols that route complex cases to appropriately scaled facilities; (c) implementing regional capacity planning to prevent redundant investment in specialized services.

*Southwest-central transition zone (Sichuan, Chongqing, Shaanxi)*: These provinces show potential for transition to high-performer status. Recommendations: (a) expand DRG payment coverage from pilot cities to provincial-wide implementation; (b) strengthen quality monitoring systems to ensure that efficiency gains do not compromise the improved undesirable output indicators; (c) develop regional medical centers of excellence to drive technological progress.

##### 5.2.2.2 Quality-safety-burden balance mechanisms

For moderate performers, the key challenge is maintaining the balance between economic efficiency and social benefits. Provincial authorities should: (a) establish TFP dashboards that separately track traditional and improved indicators, enabling real-time monitoring of the quality-efficiency trade-off; (b) implement pay-for-performance mechanisms that reward hospitals for simultaneous improvements in service volume and undesirable output reduction; (c) create patient advocacy mechanisms to ensure that efficiency improvements do not translate into increased financial burden or compromised safety.

#### 5.2.3 Recommendations for high performers

The seven high-performing provinces (Beijing, Tianjin, Shanghai, Shanxi, Xinjiang, Hubei, Liaoning) should serve as regional leaders for technology diffusion and best practice dissemination:

*Eastern coastal provinces*: Establish the Yangtze River Delta Healthcare Efficiency Alliance to: (a) standardize DRG payment methodologies across provincial boundaries; (b) develop shared quality improvement protocols that have proven effective in reducing undesirable outputs; (c) create technology transfer mechanisms that allow western provinces to benefit from eastern innovations without duplicative investment.

*Pearl river delta*: Leverage proximity to Hong Kong and Macau to: (a) pilot international best practices in hospital governance and efficiency management; (b) develop cross-border quality certification systems; (c) establish medical tourism quality standards that incentivize continuous improvement.

*Beijing-Tianjin-Hebei region*: Coordinate regional healthcare planning to: (a) optimize resource allocation across the metropolitan area; (b) implement unified quality and safety standards; (c) develop shared emergency response capabilities that proved critical during the COVID-19 pandemic.

#### 5.2.4 System-wide policy recommendations

##### 5.2.4.1 Refine the evaluation and management mechanism for hospital TFP

Establish a comprehensive TFP evaluation mechanism that incorporates medical quality, patient burden, and safety, moving beyond the traditional emphasis on quantity, profit, and input. Specifically, the National Health Commission should: (a) mandate annual TFP reporting using the improved indicator system for all tertiary hospitals; (b) incorporate TFP performance into hospital leadership evaluation criteria; (c) establish minimum TFP thresholds for hospital accreditation renewal, with differentiated standards by region to account for environmental constraints identified in Stage 2 of the DEA model.

Optimize hospital governance structures by granting greater autonomy and decision-making power, establishing effective accountability and incentive mechanisms to align operational goals with social objectives. This should include: (a) performance-based budget allocation that rewards hospitals achieving simultaneous improvements in traditional and improved TFP measures; (b) decentralized procurement authority for hospitals demonstrating responsible technology adoption practices; (c) mandatory public disclosure of TFP indicators to enable patient choice and market-based accountability.

##### 5.2.4.2 Strengthen government regulation

Implement economic regulations—such as price controls, market entry rules, and quality standards—and social regulations—including accessibility, safety, and public welfare requirements—to guide hospitals toward public health goals. Based on the empirical findings, regulatory priorities should include: (a) price caps for high-technology diagnostic procedures in provinces exhibiting suspected supplier-induced demand patterns; (b) mandatory second-opinion requirements for elective surgeries in DRG-exempt categories; (c) quality-adjusted service volume targets that prevent hospitals from achieving efficiency gains through undesirable output increases.

Introduce incentive-based regulations to encourage healthcare providers to prioritize patient health and social value, supporting the realization of “Healthy China” strategic objectives. The three-stage DEA results suggest that environmental factors significantly influence TFP; therefore, incentive structures should: (a) adjust for regional GDP per capita and urbanization differences to ensure fair comparison; (b) provide transition support for provinces facing structural disadvantages; (c) reward convergence toward national best practices rather than absolute performance levels.

##### 5.2.4.3 Enhance hospital TFP through coordinated technology management

Coordinate the introduction and use of medical equipment. The empirical analysis reveals that the relationship between technological progress and TFP is complex and region-specific. Rather than uniformly restricting or encouraging technology adoption, policy should: (a) require health technology assessment for equipment acquisitions in provinces with negative techch, with assessment criteria including projected impact on undesirable outputs; (b) establish regional equipment sharing networks in provinces with positive techch but limited access; (c) create technology adoption guidelines that distinguish between efficiency-enhancing and demand-inducing technologies.

Conduct regular assessments of regional hospital TFP. Periodic evaluation enables regulators and hospital managers to identify weaknesses, address systemic gaps, and dynamically adjust policies to promote balanced interregional development of medical services. Building on this study’s methodology, the assessment framework should: (a) employ the three-stage DEA-Malmquist approach to ensure comparability across regions and time periods; (b) report decomposed indices (techch, pech, sech) to enable targeted interventions; (c) track both traditional and improved TFP measures to monitor the quality-efficiency balance.

## Data Availability

The datasets presented in this study can be found in online repositories. The names of the repository/repositories and accession number(s) can be found in the article/Supplementary material.

## Footnotes

1 [Reviewer 2 *Point 10*] Thank you for this important clarification. We have explicitly stated the specific type of Malmquist index used (e.g., Global Malmquist) in the methodology section to ensure clarity in our panel data analysis.

2 [Reviewer 2 *Point 11*] Thank you for the suggestion. We have added a brief justification.
